# Chronic Granulomatous Disease: an Updated Experience, with Emphasis on Newly Recognized Features

**DOI:** 10.1007/s10875-022-01294-6

**Published:** 2022-06-13

**Authors:** Zacharoula Oikonomopoulou, Stanford Shulman, Marilyn Mets, Ben Katz

**Affiliations:** 1 Division of Infectious Diseases, Ann & Robert H Lurie Children’s Hospital of Chicago, 225 E Chicago Ave., Box 20, Chicago, IL 60611 USA; 2 Department of Pediatrics, Northwestern University Feinberg School of Medicine, Chicago, USA; 3 Department of Ophthalmology, Northwestern University Feinberg School of Medicine, Chicago, USA

**Keywords:** Chronic granulomatous disease, contiguous gene deletion syndrome, retinitis pigmentosa, Unfavorable Lyonization, basidiomycete pneumonia

## Abstract

**Purpose:**

Chronic granulomatous disease (CGD) is an uncommon, inborn error of immunity. We updated our large, single-center US experience with CGD and describe some newly recognized features.

**Methods:**

We retrospectively reviewed 26 patients seen from November 2013 to December 2019. Serious infections required intravenous antibiotics or hospitalization.

**Results:**

There were 21 males and 5 females. The most frequent infectious agents at presentation were aspergillus (4), serratia (4), burkholderia (2), *Staphylococcus aureus* (2), and klebsiella (2). The most common serious infections at presentation were pneumonia (6), lymphadenitis (6), and skin abscess (3). Our serious infection rate was 0.2 per patient-year from December 2013 through November 2019, down from 0.62 per patient-year from the previous study period (March 1985–November 2013). In the last 6 years, four patients were evaluated for human stem cell transplantation, two were successfully transplanted, and we had no deaths. Several patients had unusual infections or autoimmune manifestations of disease, such as pneumocystis pneumonia, basidiomycete/phellinus fungal pneumonia, and retinitis pigmentosa. We included one carrier female with unfavorable Lyonization in our cohort.

**Conclusion:**

We update of a large US single-center experience with CGD and describe some recently identified features of the illness.

## Introduction 

In 1932, it was discovered that neutrophils consume large amounts of oxygen during phagocytosis; this process was termed the “respiratory burst” [1]. Fatal granulomatous disease of childhood was first described in 1959 [2]. By 1967, it was understood that this disease was due to an inability of polymorphonuclear leukocytes to kill ingested bacteria and was thought to be exclusively X-linked; the diagnostic nitro blue tetrazolium test was also described that year and the disease was renamed chronic granulomatous disease (CGD)[3]. The first cases of autosomal recessive CGD were described in 1983 [4].

The disease is estimated to occur in about 1/100,000–1/200,000 births [5–9], with the incidence differing between ethnic groups [8], perhaps related to differences in consanguinity between different ethnic groups. Patients with CGD have defects in NADPH oxidase 2, whose structure was characterized in the late 1980s [10]. The gp91-phox defect is inherited in an X-linked manner, while the p22-phox, p40-phox, p47-phox, and p67-phox defects are inherited in an autosomal recessive manner.

The X-linked (XL) form is generally more severe, presents earlier in life, and has a higher mortality [6, 9, 11–13]. Because of the defects in NADPH oxidase, phagocytes from patients with CGD are able to ingest pathogens but are unable to mount the respiratory burst of hydrogen peroxide needed to kill them. Catalase-positive bacteria and fungi such as staphylococci, serratia, burkholderia, and aspergillus are particularly important pathogens in patients with CGD because they express some catalase and thus can neutralize the small amounts of hydrogen peroxide that phagocytes produce by mechanisms other than NADPH oxidase [6, 9, 11–14].

The most common types of infection seen in patients with CGD are pneumonia, lymphadenitis, and abscesses. As with many immune deficiencies, immunological dysregulation can also be seen, the most common syndromes of which are colitis and obstructive granulomatous lesions [6, 9, 11–13]. A less common inflammatory syndrome seen with CGD is chorioretinitis, which was first described in CGD in 1965 [15]. At first, these lesions were thought to be inactive, but since then, it has been shown that these lesions can be active and lead to vision loss [16].

Morbidity has been reported as 0.26–1.1 severe infections per patient year [11–13, 17]. Mortality in patients with CGD has steadily declined over time, due to antimicrobial prophylaxis, therapy with interferon gamma and stem cell transplantation, and mortality has generally been higher for patients with the XL form than the autosomal recessive (AR) form [6–9].

Treatment often consists of antibacterial prophylaxis with trimethoprim-sulfamethoxazole (T/S), antifungal prophylaxis with an azole (usually itraconazole [18]), and immunotherapy with interferon gamma, all of which have been shown to reduce infections in CGD [19–21]. Antibacterial prophylaxis with TS was the first successful therapy implemented for patients with CGD and became standard in the 1980s based on retrospective analyses [19, 20]. Randomized, placebo-controlled trials followed for interferon gamma [21] and itraconazole [18]. Bone marrow or stem cell transplantation can be curative [22, 23], while gene therapy (reviewed in [24]) remains under investigation as a curative therapy.

We previously reported on our large single-center experience through November 2013 [25]. We will now update that report and better describe some of the infectious and non-infectious complications seen in patients with CGD.

## Materials and Methods

This was a retrospective review of 26 patients with CGD followed at the Ann & Robert H. Lurie Children’s Hospital of Chicago (formerly Children’s Memorial Hospital) from December 2013 through November 2019. Patients followed at our hospital from March 1985 to November 2013 with CGD were reviewed previously [25]. All available paper and electronic medical records were searched. This study was approved by the Institutional Review Board of the Stanley Mann Research Institute of the Ann & Robert H Lurie Children’s Hospital of Chicago. Informed consent was not deemed necessary since this study consisted entirely of retrospective chart review.

The diagnosis of CGD was suspected based on clinical features and was subsequently confirmed by immunological and/or genetic studies, as described in our previous report [25]. Earlier patients were diagnosed using the nitro blue tetrazolium test (used since the mid-1960s [3]), later patients via flow cytometry [26].

Serious infections were defined as those related to CGD that required parenteral antibiotics and/or inpatient hospitalization, as in our previous report [25]. Charts were reviewed by at least two physicians (ZO and BK) who agreed with these assignments in every case. Patients underwent eye examinations by an ophthalmologist (MM).

Genetic data on 11 subjects were previously reported [25]. Recently, 5 additional patients have been sequenced at Gene Dx (Gaithersburg, MD). When genotyping data were unavailable, we attempted to assess whether the CGD was XL or AR based on gender, pedigree, and the dihydrorhodamine assay [27], although currently, all patients are being diagnosed genetically due to a program sponsored by Horizon Pharma. Genetic sequencing for the retinitis pigmentosa GTPase Regulator (RPGR) gene was performed at the Kellogg Eye Center of the University of Michigan.

Comprehensive eye examinations, including visual acuity measurements, pupillary examination, extraocular muscle assessment, cyclopeged refraction, and examination by slit-lamp and indirect ophthalmoscopy, were performed on both eyes when possible. Fundus photos were taken at the Northwestern Memorial Hospital Ophthalmology Retina Laboratory.

## Results

### Infections

There were 26 patients with CGD (80% males) being followed as of Nov. 2019, with a median age of 13 years (range 3–35 years; see Table 1). Since our last report [25], we excluded the 3 patients who died, 1 patient moved away, and we made 3 new diagnoses. Eighteen patients were XL and 8 were AR; sixteen of these patients were diagnosed genetically (see below), the rest were diagnosed based on gender, pedigree, and the dihydrorhodamine assay [27] as explained above. Similar to our previous report [25], nearly all patients not transplanted (17/23) were maintained on an azole (usually itraconazole [18]), trimethoprim-sulfamethoxazole and interferon-gamma; 5 patients did not tolerate interferon-gamma and one patient, the X-linked carrier with unfavorable Lyonization, was placed only on antibacterial and antifungal prophylaxis after consultation with the NIH.

**Table 1:** Patients with CGD. Those diagnosed within weeks of birth were diagnosed due to the presence of a sibling with CGD. On a few patients, the exact oxidative index was not reported, just the interpretation, and the DHR was sometimes used to assess whether the CGD was XL or AR as explained in. [27]

Patient	Sex	Age (current/ at diagnosis)	Inheritance, genetics (if known) Protein/gene affected (if known)	HSCT?	Medical therapy (1 = trimethoprim/ sulfamethoxazole, 2 = azole, 3 = interferon gamma)	Recent oxidative burst (normal oxidative index > 30)
1	M	7 y / 6 mos	XL	No	1, 2, 3	1.0
2	M	16 y / 5 mos	XL	Yes	No longer applicable	No longer applicable
3	M	13 y / 2 mos	XL	No	1, 2, 3	1.1
4	M	19 y / 7 y	AR *NCF1*:c.75_76delGT, homozygous p47 phox	No	1, 2, 3	2.3
5	M	15 y / 11 y	AR *NCF1*:c.75_76delGT, homozygous p47 phox	No	1, 2, 3	5.6
6	M	23 y / 11 y	AR	Evaluated—not a candidate	1, 2	2.5
7	M	13 y /16.5 mos	XL gp91-phox/*CYBB*	Transplant-ed elsewhere	No longer applicable	No longer applicable
8	F	25 y / 12 d	AR *NCF2*:c.1171_1175del p.Lys391Glufs*9 P67 phox	No	1, 2, 3	1.2
9	F	33 y / 5 y	AR *NCF2*:c.1171_1175del p.Lys391Glufs*9 P67 phox	No	1, 2, 3	None
10	M	7 y / 1 y	XL	No	1, 2, 3	0.9
11	M	35 y / 4.5 y	XL *CYBB*: c.C1028del gp91 phox	No	1, 2, 3	"consistent with CGD"
12	M	28 y / 4 y	XL *CYBB*:c.169dup p.Ala57Glyfs*46 gp91-phox	No	1, 2	1.0
13	M	14 y / 15 wks	XL *CYBB*: c.456 T > A, p.Y152*, hemizygous) gp91-phox	No	1, 2	1.1
14	M	21 y / 5 y	XL *CYBB*: c.469 C > T, p.R157*, hemizygous gp91-phox	Evaluated—not a candidate	1, 2, 3	1.2
15	F	26 y / 12 y	AR p47 phox/ *NCF1*	No	1, 2, 3	"consistent with CGD"
16	M	28 y / 8 y	XL gp91-phox/*CYBB*	No	1, 2, 3	None
17	M	18 y / 9 mos	XL	No	1, 2	9.4
18	M	21 y / 3.5 y	XL gp91-phox/ *CYBB*	No	1, 2	6
19	M	10 y / 8 mos	XL	No	1, 2, 3	1.2
20	M	8 y / 9 mos	XL *CYBB*: c.676 C > T, p.R226*, hemizygous gp91-phox	No	1, 2, 3	"consistent with CGD"
21	M	5 y / 4 mos	XL *CYBB*: c.141 + 5 G > T, hemizygous gp91-phox	No	1, 2, 3	0.7
22	M	26 y / 4 y	XL *CYBB*:c.1335 C > A p.Cys445* gp91-phox	No	1, 2, 3	None
23	M	10 y / 10 mos	XL gp91-phox/*CYBB*	No	1, 2, 3	0.9
24	M	13 y / 17 mos	AR	Yes	No longer applicable	No longer applicable
25	F	12 y / 4 y	AR	No	1, 2, 3	“Consistent with autosomal recessive CGD”
26	F	14 y / 8 y	XL carrier with 8–10% unfavorable lyonization	No	Cephalexin, 2	8–10% of the stimulated granulocytes display an oxidative burst of 797; the rest display an oxidative burst of 3

**Fig. 1:**
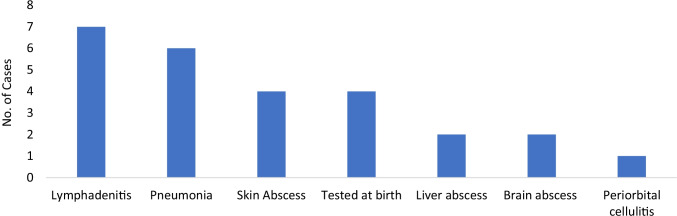
Clinical presentation at CGD diagnosis

Figure 1 depicts the clinical syndrome with which our patients presented, combining data from the previous 6 years with our initial data [25]. Lymphadenitis and pneumonia at 23% each were the most common clinical syndromes at presentation.

**Fig. 2:**
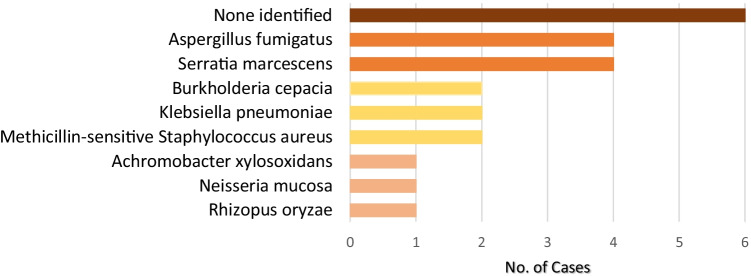
Bacterial and fungal isolates at diagnosis

Serious infections were defined as they were in our previous report — any infection that required hospitalization or therapy with intravenous antibiotics [25]. There have been 23 serious infections among the 26 patients between 2013 and 2019, yielding a rate of 0.2 per patient-year. Figure 2 depicts the known bacterial and fungal etiologies of these infections, again combining data from the previous 6 years with our initial data [25]. As reported in the literature for patients with CGD [4–8, 10, 11], the five most common infectious agents identified were aspergillus and serratia at 15% each, and burkholderia, klebsiella, and *Staphylococcus aureus* at 8% each. In 23% of cases of presumed serious infection, no pathogen was identified.

#### *Unusual Infections in Two of Our Recent Patients from 2013 to 2019* (see Table 1)

One of our patients (Patient 14), a 19-year-old male at the time, presented in July 2017 with cough and left pleuritic chest pain for 2 weeks, without fevers. He had been evaluated for transplant and deemed not a candidate because of lack of donors and previous organ damage (kidney and heart). He missed 2 weeks of IFN-γ and voriconazole prophylaxis due to insurance issues. He was initially treated with levofloxacin for 2 weeks with mild symptomatic improvement. Chest CT scan was most significant for a large wedge-shaped consolidative opacity with surrounding ground glass attenuation and air bronchograms in the posterior left lower lobe. He underwent a lung biopsy via Interventional Radiology, the fungal culture of which grew a mold that was identified as a basidiomycete/phellinus species, a rare cause of lung and other infections in patients with CGD [28]. He was initially treated with IV ambisome followed by oral posaconazole. However, after about 2 months of oral posaconazole in the setting of therapeutic posaconazole levels, his cough returned and his chest CT scan progressed. In consultation with the NIH, he was referred for a lung wedge resection for debulking of infection that he tolerated well. He received a year of intravenous ambisome and remains well on oral posaconazole with therapeutic levels. He was not a candidate for transplantation due to the lack of a suitable match and pre-existing organ damage.

A second patient (Patient 21) was referred to us after presenting at 4 months of age at another institution with pneumocystis pneumonia. He was diagnosed following a bronchoalveolar lavage during a hospitalization for a right middle lobe pneumonia that did not respond to clindamycin and cefdinir. He had a negative evaluation for other immunodeficiencies, including HIV. He was treated with IV trimethoprim-sulfamethoxazole followed by oral treatment and then prophylactic doses of the same. [29]

### Inflammatory Complications

No additional patients in our cohort developed CGD colitis or eosinophilic cystitis in the last six years. We have previously reported our two patients with eosinophilic cystitis who had recurrent episodes and were treated with prolonged, tapering courses of oral steroids and the ten patients (38.4%) with CGD-related colitis [25].

**Fig. 3:**
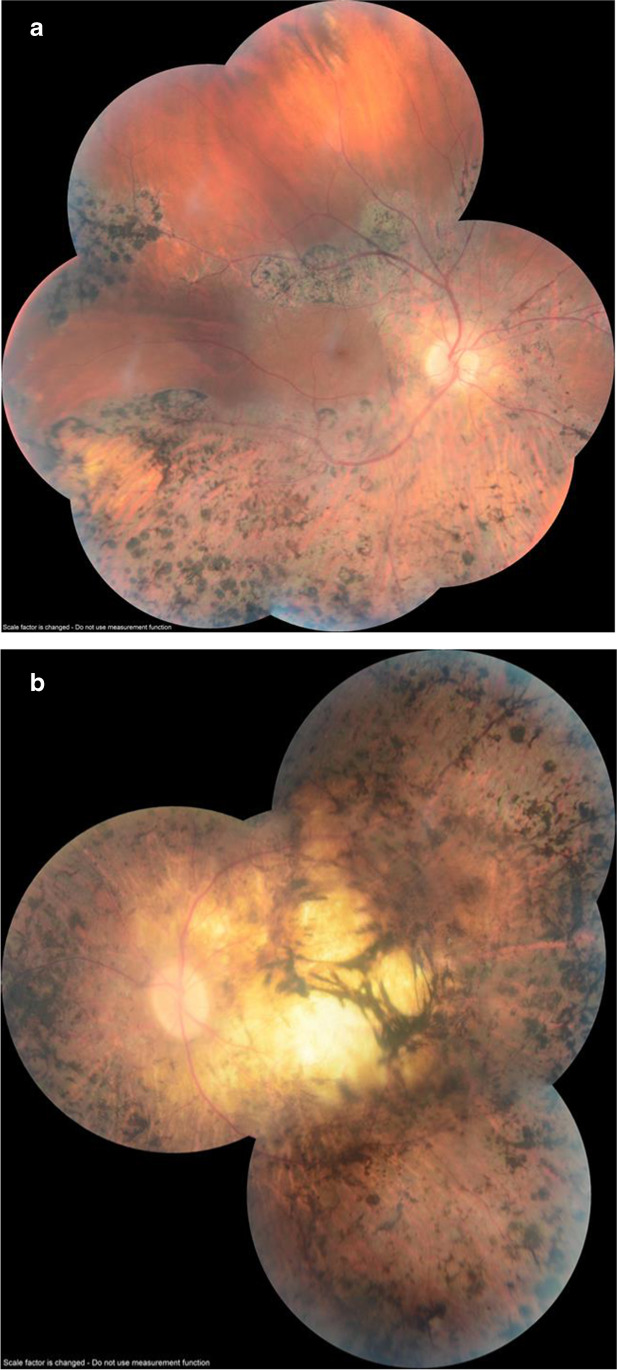
**A** Changes suggestive of retinitis pigmentosa, with migration of the retinal pigment epithelium and pigment clumping (right eye of Patient 11). **B** Changes suggestive of retinitis pigmentosa, macular involvement, optic atrophy, vessel narrowing, and generalized migration of the retinal pigment epithelium in the macular area (left eye of Patient 11)

In this report, we summarize the ophthalmologic pathology seen in our population of patients with CGD. Through 2019, 14 patients with CGD (12 of whom were X-linked) were screened by an Ophthalmologist (MM). Significant eye findings were found in three (21%); three had chorioretinitis, two of whom also had retinitis pigmentosa (RP). One of these patients had decreased visual acuity, and 2 had a history of orbital cellulitis. All ocular abnormalities were seen in patients with XL CGD. See Fig. 3 for photos from both eyes of Patient 11 with RP. Patient 11 is the only patient in our current cohort with RP; the other two patients died prior to 2013. Patient 11 has had unchanged serial visual fields for many years with no light perception in the left eye and 20/15 corrected vision in the right eye. Before being diagnosed with chorioretinitis, he also was diagnosed with CGD-related colitis.

The RP3 gene was sequenced in the two patients with RP because this region is proximal to the XL CGD locus. Neither had a mutation associated with the RP3 gene. RP2 was not sequenced.

Recently, we identified one X-linked carrier (Patient 26) with unfavorable Lyonization (only 8–10% of her neutrophils had inactivation of the affected X-chromosome, leading to a normal oxidative burst in only those neutrophils; see Table 1) who also has ornithine transcarbamalase deficiency (OTCD, which manifests as metabolic crises due to hyperammonemia), and which is also associated with CGD due to a contiguous gene deletion syndrome [30]. Because she was at increased risk for infection [31], she was begun on antibacterial and antifungal prophylaxis. Finally, one patient (patient 20) developed non-cirrhotic portal hypertension with liver fibrosis, another reported complication of and a bad prognostic sign for patients with CGD [32], although he is well currently.

### Genetic Testing

Due to a recent program sponsored by Horizon Pharma, we have been able to obtain genetic testing on 5 additional patients not previously sequenced [25], bringing our totals to 11 with defects in gp91-phagocyte oxidase (phox; XL), 3 with a defect in p47-phox (AR) and 2 with a defect in p67-phox (AR). See Table 1 for additional genetic details.

### Hematopoetic Stem Cell Transplantation and Gene Therapy

We discuss hematopoetic stem cell transplantation (HSCT) with all of our patients. From 2013 to 2019, four additional patients were referred for stem cell transplantation and one was transplanted elsewhere (Patient 7). Two have been successfully transplanted here, mainly for unremitting CGD-associated colitis, and are doing well, with resolution of their CGD and CGD-related colitis. One (Patient 2) was a mismatched unrelated transplant. The second patient (Patient 24) had a matched, related transplant. The third patient (Patient 6) was deemed not a HSCT candidate due to lack of a suitable match and pre-existing organ damage, and has been registered in a gene therapy trial. The fourth patient (Patient 14) was also not a HSCT candidate as previously discussed. Perhaps because our first transplant candidate died [25] there may have been some skittishness on the part of the patients and the physicians in enthusiastically embracing transplantation at our institution initially. During the years of this study, therapeutic dose monitoring of busulfan and reduced intensity conditioning have improved outcomes for HSCT in patients with CGD. [33]

## Discussion

CGD is a rare inborn error of immunity that predisposes patients to infections with mainly catalase-positive bacteria and fungi. In the USA, over 90% of patients with CGD are on prophylactic antibiotics (usually trimethoprim-sulfamethoxazole), about 70% are on prophylactic antifungals, but only about 35% are on interferon gamma, likely due to intolerance (which has not been a major problem in our patients), cost or lack of access. In Europe, interferon gamma is used less commonly, probably because non-randomized European data have suggested less efficacy [7].

CGD has the highest prevalence of fungal infections among inborn errors of immunity (20–40%) [34]. The most common fungal isolate in patients with CGD is aspergillus, as was seen in our cohort [25]. The use of anti-fungal prophylaxis has led to the emergence of infections with non-aspergillus species. We report an unusual case of a basidiomycosis infection in one of our patients in the last six years, who required a lobectomy for debulking of infection and remains on medical therapy (> 2 years).

From March 1985 to November 2013, our rate of serious infections was 0.62 per patient-year [25]. From December 2013 through November 2019, our rate was 0.2 per patient-year. It is unclear why our serious infection rate has decreased, although increasing clinical experience, earlier age at diagnosis, better and less invasive diagnostic testing, and curing some children via stem cell transplant probably all played a role.

We previously reported that ten patients from our cohort (38.4%) developed CGD-related colitis [25]. No additional patient has developed CGD- related colitis from 2013 to 2019.

Three patients of 14 examined (21%) developed chorioretinitis, two of whom also had retinitis pigmentosa (RP). Previous studies have had rates of 13–24% for ocular abnormalities in patients with CGD [35, 36]. There are multiple genetic mutations responsible for RP. The most common is mutation in the RP3 gene, but other mutations such as RP2 comprise 10–26% of cases [37]. There is no phenotypic difference between the RP2 and RP3 mutant genotypes [38], so it is possible that our two patients with CGD and RP had an RP2 (or other) mutation in the absence of an RP3 mutation.

OTCD and RP are thought to develop in CGD due to contiguous gene deletion syndromes [30], and all 3 defects have also been described in a single patient due to a contiguous gene deletion as well [39]. RP can also be seen in X-linked carriers of CGD [35], as is the case with other autoimmune manifestations of CGD [40].

CGD-associated colitis and many active infections will resolve after HSCT. Two of our recently transplanted patients with CGD had resolution of both their CGD and colitis.

In conclusion, our cohort continues to do relatively well. Most of our patients continue to be maintained on antibacterial prophylaxis (usually with trimethoprim-sulfamethoxazole), antifungal prophylaxis (usually with itraconazole [17]) and immunomodulation with interferon gamma. Several have been transplanted and have done well. We have had no deaths in the past six years and our serious infection rate has decreased. We continue to see unusual infections and autoimmune manifestations in this group of patients. We continue to promote HSCT.

## Data Availability

The data sets generated during the current study are available from the corresponding author on reasonable request.
