# Correction to: Chronic Granulomatous Disease: an Updated Experience, with Emphasis on Newly Recognized Features

**DOI:** 10.1007/s10875-022-01370-x

**Published:** 2022-09-26

**Authors:** Zacharoula Oikonomopoulou, Stanford Shulman, Marilyn Mets, Ben Katz

**Affiliations:** 1 Division of Infectious Diseases, Ann & Robert H Lurie Children’s Hospital of Chicago, 225 E Chicago Ave., Box 20, Chicago, IL 60611 USA; 2 Department of Pediatrics, Northwestern University Feinberg School of Medicine, Chicago, USA; 3 Department of Ophthalmology, Northwestern University Feinberg School of Medicine, Chicago, USA

**Correction to: Journal of Clinical Immunology**

**https://doi.org/10.1007/s10875-022-01294-6**

The article "Chronic Granulomatous Disease: an Updated Experience, with Emphasis on Newly Recognized Features", written by Oikonomopoulou, Z., Shulman, S., Mets, M. and Katz, B., was originally published electronically on the publisher’s internet portal on 13 June 2022 without open access. With the author(s)’ decision to opt for Open Choice the copyright of the article changed on 6 September 2022 to © © The Author(s) 2022 and the article is forthwith distributed under a Creative Commons Attribution 4.0 International License, which permits use, sharing, adaptation, distribution and reproduction in any medium or format, as long as you give appropriate credit to the original author(s) and the source, provide a link to the Creative Commons licence, and indicate if changes were made. The images or other third party material in this article are included in the article's Creative Commons licence, unless indicated otherwise in a credit line to the material. If material is not included in the article's Creative Commons licence and your intended use is not permitted by statutory regulation or exceeds the permitted use, you will need to obtain permission directly from the copyright holder. To view a copy of this licence, visit http://creativecommons.org/licenses/by/4.0/

The original version has been corrected.

